# Supplementary material for: Micronutrient Deficiencies in Heart Failure and Relationship with Exocrine Pancreatic Insufficiency
Source: Nutrients. 2024 Dec 27;17(1):56. doi: 10.3390/nu17010056 (PMC11723028; doi:10.3390/nu17010056)
Supplement: Supplementary file 1 [file nutrients-17-00056-s001.zip › nutrients-3361040-supplementary.pdf]

SUPPLEMENTARY MATERIAL

Supplementary Table S1: Linear regression vitamin A, D, E, zinc and selenium

**S1.1 Univariate linear regression vitamin A (retinol)****					
	B	*S.E.*	*β*	*95% CI*	*p-value*
**Age**	0.002	0.01	0.19	-0.001, 0.004	0.16
**Weight**	0.000	0.001	0.06	-0.002, 0.003	0.67
**BMI**	0.001	0.004	0.04	-0.007, 0.009	0.76
**Duration of HF***	0.01	0.29	-0.03	-0.052, 0.064	0.83
**NT-proBNP***	0.19	0.04	0.07	-0.051, 0.088	0.59
**FE-1**	0.000	0.000	0.08	0.000, 0.000	0.55
**Amylase***	0.09	0.07	0.17	-0.050, 0.238	0.20
**Lipase***	0.05	0.07	0.10	-0.086, 0.182	0.77
**LVEF**	-0.001	0.001	-0.06	-0.003, 0.002	0.65
**Cardiac index***	-0.06	0.18	-0.06	-0.427, 0.306	0.74
**S1.2 Univariate linear regression vitamin D (D3)***					
**Age**	0.01	0.003	0.21	-0.001, 0.010	0.13
**Weight**	-0.01	0.002	-0.29	-0.010, -0.001	**0.03**
**BMI**	-0.02	0.01	-0.37	-0.040, -0.007	**0.01**
**Duration of HF***	0.6	0.06	0.12	-0.069, 0.187	0.34
**NT-proBNP***	0.04	0.08	0.07	-0.117, 0.192	0.63
**FE-1**	0.00	0.00	0.08	-0.001, 0.001	0.57
**Amylase***	0.19	0.16	0.16	-0.231, 0.516	0.23
**Lipase***	0.01	0.15	0.01	-0.295, 0.307	0.97
**LVEF**	0.11	0.06	0.23	-0.015, 0.235	0.08
**Cardiac index***	0.59	0.44	0.23	-0.97, 1.483	0.18

**S1.3 Univariate linear regression vitamin E (α-tocopherol)**					
**Age**	0.20	0.08	0.30	0.033, 0.0369	**0.02**
**Weight**	-0.5	0.07	-0.09	-0.188, 0.093	0.50
**BMI**	-0001	0.27	-0.00	-0.538, 0.523	0.98
**Duration of HF***	-1.72	1.93	-0.12	-5.582, 2.138	0.38
**NT-proBNP***	0.80	2.43	0.05	-3.898, 5.488	0.73
**FE-1**	0.01	0.01	0.05	-0.020, 0.030	0.70
**Amylase***	6.34	4.91	0.17	-3.44, 16.24	0.20
**Lipase***	-0.08	4.58	-0.00	-9.261, 9.098	0.97
**LVEF**	-0.05	0.09	-0.7	-0.229, 0.131	0.59
**Cardiac index***	2.33	13.36	0.03	-24.84, 29.50	0.86
**S1.4 Univariate linear regression selenium**					
**Age**	0.13	0.14	0.13	-0.142, 0.403	0.34
**Weight**	0.001	0.11	0.002	-0.217, 0.220	0.99
**BMI**	-0.07	0.41	-0.41	-0.881, 0.744	0.87
**Duration of HF***	-5.15	2.91	-0.232	-10.98, 0.675	0.08
**NT-proBNP***	-1.19	3.58	-0.05	-8.365, 5.977	0.74
**FE-1**	0.02	0.02	0.13	-0.19, 0.056	0.34
**Amylase***	-6.72	7.49	-0.12	-21.73, 8.285	0.37
**Lipase***	-6.43	6.91	-0.12	-20.28, 7.420	0.36
**LVEF**	-0.11	0.14	-0.11	-0.388, 0.161	0.41
**Cardiac index***	-12.31	19.79	-.011	-52.63, 28.01	0.54
**S1.5 Univariate linear regression zinc**					
**Age**	-0.03	0.03	-0.16	-0.087, 0.021	0.23
**Weight**	0.02	0.02	0.13	-0.022, 0.065	0.32

**BMI**	0.05	0.08	0.09	-0.109, 0.214	0.52
**Duration of HF***	-0.24	0.60	-0.06	-1.433, 0.952	0.69
**NT-proBNP***	-0.63	0.71	-0.12	-2.025, 0.791	0.38
**FE-1**	-0.001	-0.004	-0.02	-0.008, 0.007	0.81
**Amylase***	1.76	1.49	0.16	-1.222, 4.733	0.24
**Lipase***	-0.48	1.39	-0.05	-3.261, 2.302	0.73
**LVEF**	-0.01	0.03	-0.05	-0.065, 0.045	0.71
**Cardiac index***	-0.37	0.67	0.10	-1.724, 0.990	0.59

Legen table S1: *β: regression coefficient, S.E.: standard error, OR: odds ratio, CI: confidence interval. *: logarithmic transformation, ** logarithmic transformation + 1. Abbreviations: HF :heart failure, NT-proBNP: N-terminal pro-B-type natriuretic peptide, FE-1: faecal elastase 1, LVEF: left ventricular ejection fraction.*
